# Age-Stratified Analysis of Social Environmental Drivers of Depression Among Chinese Young People Aged 10–24 Years

**DOI:** 10.62641/aep.v54i3.2236

**Published:** 2026-06-15

**Authors:** Xiongwei Xiang, Mingyu Fu, Jianing Wu, Ruiyang Chen, Xionggen Xiang, Yao Zhou, Yongchun Li, Weiwei Gao, Shuji Li

**Affiliations:** ^1^College of Physical Education, Hunan Normal University, 410081 Changsha, Hunan, China; ^2^Department of Physiology, School of Basic Medical Sciences, Southern Medical University, 510515 Guangzhou, Guangdong, China; ^3^Senior Three Grade Group, YK Pao School, 200000 Shanghai, China; ^4^School of Arts and Humanities, Tung Wah College, Hong Kong, China; ^5^R&D Center, Hunan Weishi Biotechnology Research Institute, 410000 Changsha, Hunan, China; ^6^Traditional Chinese Medicine Department, Nanfang Hospital, Southern Medical University, 510515 Guangzhou, Guangdong, China; ^7^Guangdong–Hong Kong–Macao Greater Bay Area Center for Brain Science and Brain-Inspired Intelligence, Southern Medical University, 510515 Guangzhou, Guangdong, China

**Keywords:** depression, adolescent, social determinants of health, longitudinal studies, mental health

## Abstract

**Background:**

This study aimed to evaluate the effect of key social environmental determinants on trends in depression burden amongst Chinese adolescents aged 10–24 years and to examine the age- and gender-specific variations and lagged effects of these factors.

**Methods:**

Nationwide data spanning 2003–2021 were collected from the Global Burden of Disease database for depression burden indicators, World Bank socioeconomic datasets and the Chinese General Social Survey for education and social metrics. The primary variables analysed were gross domestic product (GDP) per capita, higher education enrolment rate (HEER), per capita current health expenditure and urban population percentage. Descriptive statistics and Pearson correlations were used to explore variable distributions and associations. Mixed-effects regression models quantified relationships between social factors and depression burden, and autoregressive distributed lag models assessed short-term lagged effects across age groups (10–14, 15–19 and 20–24 years). Interaction terms (HEER × total public expenditure on education (TPEE); unemployment × dependency ratio) were included on the basis of theoretical and empirical support from prior studies.

**Results:**

Between 2003 and 2021, the overall burden of depression amongst young Chinese people decreased, but the 10- to 14-year age group showed a notable upward trend in disability‑adjusted life‑years since 2017. High GDP per capita, urbanisation and per capita health expenditure were significantly associated with reduced depression burden (*p* < 0.05). Conversely, increasing HEER—indicative of rising educational competition—was associated with a high disease burden, particularly amongst females aged 15–19 and 20–24 years. Short-term lagged effects revealed that the psychological burden of education competition manifested within 1 year, particularly amongst younger adolescents. Economic improvements and health investments exerted protective lagged effects. Age-stratified analyses underscored distinct vulnerability patterns: 10- to 19-year-olds were highly sensitive to family and educational support, and 20- to 24-year-olds were greatly affected by social structural pressures. Pearson correlation analysis identified significant negative associations between several social factors and depression burden. The mechanisms were explained by China-specific social contexts and data characteristics.

**Conclusions:**

This nationwide longitudinal study reveals that multidimensional social determinants exert age- and gender-specific influences on depression burden amongst young Chinese people aged 10–24 years. The findings emphasise the urgent need for stratified policies, including enhancing family and educational support for individuals under 20 years old and reducing structural social pressures on young adults. Public mental health interventions should target these modifiable social determinants to reduce the depression burden and improve well-being.

## Introduction

Depression amongst young people has emerged as a critical global public health challenge [1, 2], with its burden particularly severe in rapidly transforming societies such as China. A 2021 epidemiological survey reported that 17.5% of Chinese students aged 6–16 years exhibit mental disorders, with mood disorders being prevalent amongst those aged 12–16 years [3]. Depression, characterised by persistent sadness, interest loss and functional impairment, disrupts psychological and emotional development and has long-lasting impacts on educational attainment, workforce participation and overall quality of life [4]. Over the past two decades, China’s significant social, economic and cultural changes have restructured the developmental environment for young people, substantially altering the epidemiology and risk profiles of depression in this population [5, 6]. Rapid urbanisation, education system expansion and increasing social mobility have created new pressures and opportunities for Chinese youth and might have influenced their mental health in different ways across developmental stages. For example, adolescents often face intense academic competition and family expectations; young adults encounter additional challenges related to higher education transitions, employment and social independence. These distinct life-course transitions suggest that the social determinants of depression may vary substantially across age groups. However, most existing studies in China examined youth depression as a single population, with limited attention to age-stratified analyses of socioeconomic determinants. Therefore, understanding how these factors differentially influence depression burden across the social transition from 10 to 19 years of age may provide targeted insights for mental health prevention and policy interventions.

This study follows the World Health Organization definition of “young people” as individuals aged 10–24 years, encompassing adolescence (10–19 years) and early adulthood (20–24 years). This classification aligns with the developmental characteristics of young Chinese people during social transition—10- to 19-year-olds focus on academic and family adaptation, and 20- to 24-year-olds face transitions to employment and independent living—and ensures consistency with global public health research standards.

Mental health is profoundly shaped by the social determinants of health (SDHs) that influence conditions of birth, growth, life and work [7, 8, 9, 10]. A growing body of evidence highlights that favourable SDHs contribute to physical and mental well-being. Social environmental factors, such as family income, educational opportunities, employment status, healthcare expenditure and urbanisation—are increasingly recognised as central determinants of depression amongst young people [11]. In China, adolescents from lower socioeconomic backgrounds bear a disproportionately high burden of depression, with social support acting as a critical mediating factor [12]. Additionally, family structure and function—including parental education levels, marital stability and perceived familial support—are well-established predictors of mental health. Young people experiencing family dysfunction, divorce or low parental education levels face significantly high risks of depression [13, 14]. In urban areas undergoing rapid demographic changes, social isolation and limited access to supportive social networks exacerbate the burden of depression [15].

Depression amongst younger adolescents (10–14 years) is closely linked to educational investment and social support factors, such as public education expenditure and urban development [4]. By contrast, early adults (20–24 years) are highly sensitive to personal development indicators, including family income, higher education enrolment rate (HEER) and access to healthcare resources—factors often perceived as determinants of future stability [5]. Mounting evidence suggests that younger adolescents are especially vulnerable to changes in family dynamics, such as parental divorce or low emotional support, which can critically undermine their sense of security and belonging [13]. Conversely, early adults face heightened risk from structural social factors such as academic pressure, educational opportunities and employment prospects, which intersect with emerging adult responsibilities and future-oriented anxiety to increase depression vulnerability [16].

The increasing use of information technology amongst young Chinese people has introduced new challenges to mental health. Excessive internet use for entertainment and gaming correlates with elevated depressive symptoms, highlighting how shifts in social behaviour and recreational patterns impact psychological well-being [17]. By contrast, structured academic engagement and online learning do not exhibit such negative associations, suggesting a nuanced relationship between technology use and mental health outcomes.

Building upon existing literature, this study leverages comprehensive longitudinal datasets—including the Global Burden of Disease (GBD), World Bank and China General Social Survey (CGSS)—to systematically evaluate how multidimensional social environmental factors (economic indicators, educational opportunities, public health investments, urbanisation trends and family structure) influence depression burden amongst young Chinese people aged 10–24 years from 2003 to 2021. By employing mixed-effects regression (with year as the random effect) and lagged effect modelling, this research aims to construct an empirical framework that elucidates the direct and short-term lagged impacts of social environments on depression. The findings inform policy-relevant, age-sensitive strategies for enhancing mental health in this population.

## Materials and Methods

### Data Sources and Collection

Data were obtained from three primary sources: (1) the GBD database (https://www.thelancet.com/gbd), covering depression burden metrics (incidence, prevalence and disability‑adjusted life‑years [DALYs]) for all age groups in China from 1990 to 2021; (2) the World Bank Open Data database (https://data.worldbank.org/), providing annual macrolevel socioeconomic indicators (e.g., GDP per capita, unemployment rate, urban population percentage); and (3) CGSS, accessed via the Chinese National Survey Data Archive (http://cgss.ruc.edu.cn/), offering representative microlevel data on social and family variables (e.g., divorce ratio and middle-income ratio) collected from 2003 to 2021.

For the integration of these three databases, relevant variables were firstly extracted from each source using consistent country level identifiers (ISO codes for China) and then aligned by calendar year from 2003 to 2021—the overlapping period during which data were available across all three databases. For the GBD data, annual estimates of depression incidence, prevalence and DALYs were extracted for the age groups of interest (10–14, 15–19 and 20–24 years of age). For the World Bank data, annual macroeconomic indicators were retrieved. For CGSS, individual level responses were aggregated to annual averages to match the temporal resolution of the other datasets. All the datasets were then merged into a single panel dataset using a full join approach on country and year. Missing values were handled by linear interpolation for time series variables and by list wise deletion for cross sectional variables where interpolation was not feasible.

Depression burden was defined according to the GBD framework, which follows the diagnostic criteria for depressive disorders from the Diagnostic and Statistical Manual of Mental Disorders. The GBD framework quantifies depression using DALYs, a composite metric that sums years of life lost (YLLs) due to premature mortality and years lived with disability (YLDs), which were derived from prevalence estimates multiplied by disability weights [18]. For the GBD database, the disease burden indicators were estimated using a systematic analytical framework. Data sources included vital registration systems, verbal autopsies, censuses, household surveys, disease specific registries, health service contact data and other sources. YLDs were calculated by multiplying cause and sequelae specific prevalence estimates by disability weights derived from community surveys. YLLs were calculated by multiplying cause specific deaths by standard life expectancy at the age of death. DALYs were then summed from the YLDs and YLLs, with 95% uncertainty intervals generated from 500 draws [19]. These methods were consistently applied in earlier GBD cycles, including GBD 2010 [20].

Socioeconomic indicators from the World Bank Open Data database were compiled from the statistical systems of member countries and officially recognised international sources, applying consistent definitions to ensure cross country comparability. CGSS adopts a multistage, stratified random sampling with probability proportional to size, collecting data via standardised face to face interviews on socio demographics, health, family structure and social attitudes.

Data selection and integration followed the GBD project’s standardised methodological framework to ensure consistency, reliability and validity. The final analytical dataset included harmonised data from 2003 to 2021, the period in which comprehensive data were consistently available across the sources.

### Variable Selection and Operationalisation

Primary outcomes were defined as annual prevalence, incidence and DALYs of depression amongst young Chinese people aged 10–24 years. Independent variables included nine macrolevel social determinants from the World Bank dataset, categorised into:

• Labour: labour force participation rate (15–24 years), total unemployment rate;

• Economy: gross domestic product (GDP) per capita, gross national income (GNI) per capita;

• Healthcare: current health expenditure per capita (CHE per capita);

• Education: HEER, total public expenditure on education (TPEE);

• Urbanisation: urban population percentage; and

• Demographic: dependency ratio (non-working-age population/working-age population).

Family-level factors, including divorce ratio, total education level of respondent (TER), spouse/partner’s highest education rating (SHER) and middle-income ratio, were also extracted from CGSS.

Subjective well-being indicators were not included as primary outcomes because of insufficient evidence supporting their validity as proxies for clinical depression burden. However, the unhappiness ratio was retained as a secondary, exploratory variable to examine its statistical association with depression burden in supplementary analyses. It was not used as a primary dependent variable in the main analytical models. Given that the unhappiness ratio is not a clinically validated diagnostic measure of depression, all results involving this variable should be interpreted with caution and are intended only to provide complementary insights rather than infer clinical relevance.

### Data Processing and Missing Data Imputation

Variables were standardised to account for differences in CGSS questionnaire options across years. Categorical survey data were aggregated into macrolevel proportions to align with GBD and World Bank metrics. Missing data were imputed using multiple imputation methods appropriate for time-series data:

• Missing values for 2004, 2007, 2009, 2014 and 2016 were imputed by averaging adjacent years’ values;

• Missing data for 2019 and 2020 were estimated using polynomial regression to account for temporal trends.

Collinearity diagnostics were performed using variance inflation factor thresholds (< 5) before regression modelling, with highly correlated variables (e.g., GDP per capita and GNI per capita) handled through sequential adjustment in regression models.

### Time Series and Correlation Analysis

Annual trends in depression burden and social determinants from 2003 to 2021 were analysed using time series methods to identify long-term trends and short-term fluctuations. Pearson correlation coefficients were calculated to assess linear associations between depression burden variables and social determinants. Correlations were tested against a two-tailed null hypothesis of no significant linear relationship, with *p* < 0.05 considered statistically significant. Statistical assumptions (residual normality and homoscedasticity) were examined via Q-Q plots and residual distribution tests to ensure validity.

### Mixed-Effects Regression Modelling

A mixed-effects regression model was developed with depression incidence/prevalence/DALYs as the dependent variables, year as the random effect (to control for time-varying unobserved heterogeneity, e.g., policy changes, public health events) and selected independent variables as fixed effects to account for potential fixed and random effects. This specification aligns with macro time-series data analysis norms, as “year” better captures unobserved confounding compared with gender (a fixed categorical variable) [21].

Interaction terms were specified based on socioecological theory and prior empirical evidence. The HEER × TPEE interaction captures the competition–resource matching mechanism, whereby equitable education investment mitigates the adverse mental health effects of rising enrolment pressure [4]. The unemployment × dependency ratio interaction reflects the amplification of economic stress in high dependency households, consistent with findings that family structure moderates unemployment related risks for adolescent depression [5]. These specifications were therefore theory driven rather than arbitrary.

1. HEER × TPEE: HEER [22] is used as a proxy for the intensity of educational competition, and TPEE reflects the supply of educational resources. Although HEER primarily measures access to education, it generally indicates increased participation in the education system, which can intensify competition for limited high-quality educational resources, particularly in regions with high population density and constrained school capacity. This intensified competition is often accompanied with great academic expectations and peer pressure, thereby contributing to psychological stress. TPEE was further incorporated as an indicator of resource availability to comprehensively capture the structural context of educational competition. The interaction term (HEER × TPEE) reflects the balance between competitive pressure and resource supply. Educational resource equity can mitigate the adverse effects of competitive pressure on adolescents’ mental health [4]. Therefore, this interaction term represents a ‘competition–resource matching’ mechanism, which is critical for understanding how educational environments influence depression burden.

2. Unemployment × dependency ratio: Economic pressure from unemployment is amplified in high-dependency-ratio families (e.g., childcare costs in multichild households), whilst low-dependency-ratio families have strong buffering capacity [5]. This interaction captures family structure’s moderating role on unemployment stress, consistent with the social ecological model’s multilevel interaction hypothesis [11].

The model specification was:

Depression Incidence/Prevalence/DALYs= β_0_ + β_1_ (Dependency Ratio) + β_2_ (GDP Per Capita) + β_3_ (HEER × TPEE) + β_4_ (Divorce Ratio) + β_5_ (Middle-Income Ratio) + β_6_ (Unemployment × Dependency Ratio) + (1—Year).

### Lagged Effect Analysis

HEER, CHE and urbanisation were selected as covariates for the autoregressive distributed lag (ARDL) models. This choice was guided by their theoretical relevance to adolescent depression burden: HEER is interpreted as a proxy for educational competition, CHE per capita captures public health investment, and urbanisation indicates structural changes in social support environments. The selection is also constrained by data availability and the requirement for consistent time series coverage. Whilst these variables align with socioecological perspectives and prior literature, the absence of a fully systematic model screening procedure represents a methodological limitation, which is further acknowledged in the discussion and limitations sections.

The short- and long-term lagged effects of HEER, CHE per capita and urban population percentage on depression incidence were examined using ARDL modelling, which is suitable for analysing relationships amongst nonstationary time series variables. Prior to model estimation, the stationarity of each variable was assessed using unit root tests to ensure that the variables were integrated of order I(0) or I(1) as required for ARDL modelling [23, 24].

Lag periods of up to 3 years were considered on the basis of theoretical plausibility and data structure. The optimal lag order for each variable was determined using information criteria, including the Akaike information criterion and Bayesian information criterion, by comparing alternative model specifications. This procedure allows for the identification of the most parsimonious model whilst adequately capturing temporal dependencies. Long-run relationships amongst variables were further evaluated using the bounds testing approach to assess cointegration.

Variable inclusion in the ARDL model was guided by the socioecological framework and prior research [11, 25], where HEER represents educational structural factors shaping adolescents’ exposure to academic pressure, CHE per capita reflects public health investment as a key determinant of mental health service accessibility and urban population percentage captures broad social environmental changes (e.g., variations in social support networks and resource availability) [11]. The ARDL framework enables the simultaneous estimation of autoregressive and distributed lag components, providing a flexible approach for modelling short-term dynamics and long-term equilibrium relationships.

### Statistical Software and Significance Thresholds

All statistical analyses were conducted using R software version 4.2.1 (R Foundation for Statistical Computing, Vienna, Austria). Packages used included nlme for mixed-effects models and dynlm for ARDL analysis. All statistical tests were two-sided, and statistical significance was defined as *p* < 0.05.

## Results

### Overall Trends of Depression Burden and Social Environmental Factors

**Fig. 1.**
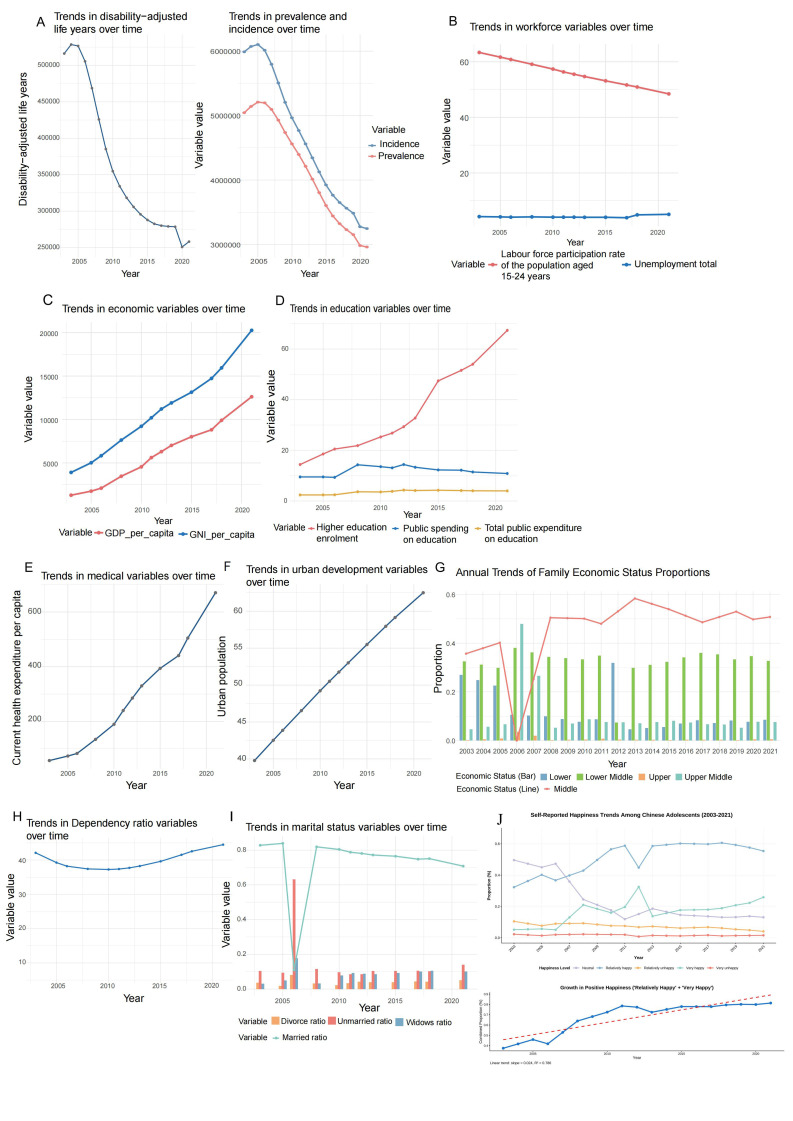
**Overall Trends of Depression Burden and Social Environmental Factors Amongst Chinese Adolescents (2003–2021)**. (A) Annual depression burden trends measured by incidence, prevalence and disability-adjusted life years (DALYs). (B) Labor indicators: labour force participation and unemployment rates for ages 15–24. (C) Economic indicators: GDP per capita and GNI per capita. (D) Education indicators include the higher education enrolment rate (HEER) and total public expenditure on education (TPEE). (E) Per capita health expenditure (CHE). (F) Urban population percentage. (G) Middle-income household proportion. (H) Dependency ratio. (I) Marriage rate. (J) Proportions of self-reported happiness and unhappiness. Data sources: GBD, World Bank, CGSS.

Between 2003 and 2021, the burden of depression amongst young Chinese people aged 10–24 peaked in 2005 and subsequently declined (Fig. 1). Incidence and prevalence decreased markedly from 2019 to 2020 and continued to decline gradually thereafter, whereas DALYs showed a slight increase after 2020. These trends are potentially related to the indirect impacts of the COVID-19 pandemic (Fig. 1A).

Labour force participation amongst individuals aged 15–24 declined steadily, whilst unemployment rates remained relatively stable (4.2%–5.8%) (Fig. 1B). Economic indicators, including GDP per capita and GNI per capita, increased consistently throughout the study period (compound annual growth rates of 8.3% and 8.1%, respectively) (Fig. 1C).

Educational indicators showed that HEER rose continuously from 15.8% in 2003 to 57.8% in 2021, whilst TEER increased sharply between 2006 and 2008 and then plateaued (Fig. 1D). CHE per capita grew exponentially (compound annual growth rate: 11.2%), and urban population percentage increased from 40.5% to 64.7% (Fig. 1E,F).

Family and demographic indicators also changed over time. The proportion of middle-income households gradually increased (with a brief decline in 2006), the dependency ratio firstly decreased and then rose, and the marriage rate declined slowly from 7.6‰ in 2003 to 5.4‰ in 2021 (Fig. 1G–I). Additionally, self-reported happiness improved progressively, with an increasing number of adolescents reporting being “relatively happy” or “very happy” (Fig. 1J).

### Correlation Analysis

Pearson correlation analysis revealed significant negative associations between social environmental factors and depression burden indicators (incidence, prevalence and DALYs) amongst Chinese adolescents. GDP per capita and urban population percentage exhibited the strongest negative correlation with DALYs (β = −0.345), and urban population percentage was negatively correlated with incidence (β = −0.406). Additionally, GDP per capita and CHE per capita showed a negative correlation with prevalence (β −0.314), all *p* < 0.05 (Table 1). Multicollinearity analysis revealed significant correlations amongst CHE per capita, GNI per capita, GDP per capita and urban population percentage (**Supplementary Fig. 1**), prompting adjustments to these variables in the subsequent regression models.

**Table 1.** **Correlation between depression burden and social environmental factors amongst Chinese adolescents (2003–2021)**.

Dependent variable	Independent variable	coefficient	*p*
Disability-adjusted life years	Unemployment	−0.188	0.045
Disability-adjusted life years	GDP per capita	−0.345	<0.001
Disability-adjusted life years	GNI per capita	−0.343	<0.001
Disability-adjusted life years	CHE per capita	−0.344	<0.001
Disability-adjusted life years	Urban population	−0.345	<0.001
Disability-adjusted life years	HEER	−0.340	<0.001
Disability-adjusted life years	TPEE	−0.296	0.001
Disability-adjusted life years	Dependency Ratio	−0.220	0.018
Disability-adjusted life years	Divorce Ratio	−0.082	0.386
Disability-adjusted life years	TER	−0.156	0.097
Disability-adjusted life years	SHER	0.096	0.308
Disability-adjusted life years	Happiness Ratio	−0.205	0.029
Disability-adjusted life years	Middle income Ratio	−0.145	0.123
Incidence	Unemployment	−0.207	0.027
Incidence	GDP per capita	−0.404	<0.001
Incidence	GNI per capita	−0.402	<0.001
Incidence	CHE per capita	−0.400	<0.001
Incidence	Urban population	−0.406	<0.001
Incidence	HEER	−0.393	<0.001
Incidence	TPEE	−0.361	<0.001
Incidence	Dependency Ratio	−0.234	0.012
Incidence	Divorce Ratio	−0.089	0.344
Incidence	TER	−0.165	0.079
Incidence	SHER	0.109	0.246
Incidence	Happiness Ratio	−0.259	0.005
Incidence	Middle income Ratio	−0.184	0.050
Prevalence	Unemployment	−0.177	0.060
Prevalence	GDP per capita	−0.314	<0.001
Prevalence	GNI per capita	−0.312	<0.001
Prevalence	CHE per capita	−0.314	<0.001
Prevalence	Urban population	−0.313	<0.001
Prevalence	HEER	−0.312	<0.001
Prevalence	TPEE	−0.262	0.005
Prevalence	Dependency Ratio	−0.212	0.024
Prevalence	Divorce Ratio	−0.079	0.400
Prevalence	TER	−0.155	0.100
Prevalence	SHER	0.098	0.298
Prevalence	Happiness Ratio	−0.174	0.064
Prevalence	Middle income Ratio	−0.122	0.196

Note: DALYs, Disability-adjusted life years; GDP, Gross domestic product; GNI, Gross national income; CHE, Per capita health expenditure; HEER, Higher education enrolment rate; TPEE, Total public expenditure on education; TER, Total education level of respondent; SHER, Spouse/partner’s highest education rating. Coefficients (β) and *p*-values from Pearson correlation analysis.

Although Table 1 shows predominantly negative correlations between several social factors and depression burden, these associations should be interpreted cautiously and do not imply direct causal relationships. Specifically, unemployment exhibited a negative correlation with DALYs and incidence, whilst the dependency ratio was negatively associated with DALYs, incidence and prevalence. The correlation between divorce ratio and prevalence was also negative but not statistically significant. Overall, these findings indicate that macrolevel social indicators are related to youth depression burden, but the patterns are complex and likely influenced by multiple contextual factors rather than reflecting direct protective effects.

**Fig. 2.**
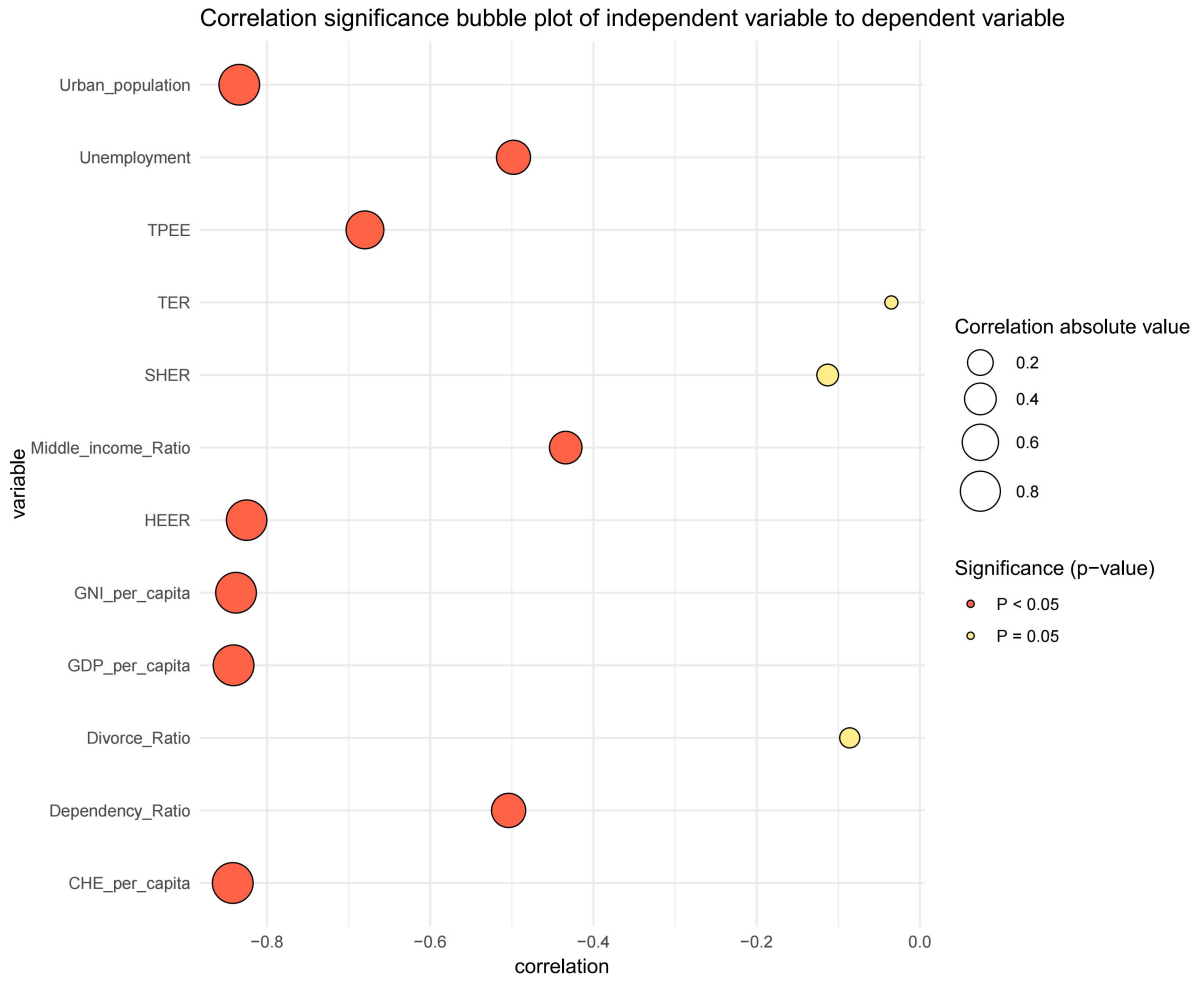
**Correlation between social determinants and mental health indicators**. Bubble plot showing the correlation coefficients between social factors and the dependent variable.Note: TPEE, Total public expenditure on education; TER, Total education level of respondent; SHER: Spouse/partner’s highest education rating; HEER, Higher education enrolment rate; GNI, Gross national income; CHE, Per capita health expenditure. Bubble size represents the absolute value of the correlation coefficient, and colour indicates statistical significance (red: *p* < 0.05; yellow: *p* = 0.05).

Correlation analysis revealed significant associations between unhappiness ratio and depression burden indicators. A high unhappiness ratio was positively correlated with increased incidence, prevalence and DALYs (all *p* < 0.05; Table 2). Further correlation analysis between social factors and unhappiness ratio showed that most variables were significantly negatively correlated with unhappiness. Specifically, all variables exhibited significant negative correlations except for divorce ratio, SHER and TER (Fig. 2; **Supplementary Table 1**). Amongst these factors, CHE per capita demonstrated one of the strongest negative correlations (β = −0.842, *p* < 0.001). These findings suggest that improvements in socioeconomic conditions—particularly increased healthcare investment—may be indirectly associated with good adolescent mental health through reductions in unhappiness. However, the associations involving the unhappiness ratio should be interpreted cautiously because of the exploratory nature of this variable.

**Table 2.** **Univariate regression analysis between unhappiness ratio and depression metrics**.

Outcome	β (regression coefficient)	Std Error	t	*p*	95% CI
Disability-adjusted life years	2.09 × 10^6^	6.27 × 10^5^	3.338	0.001	[8.50 × 10^5^, 3.33 × 10^6^]
Incidence	1.38 × 10^7^	3.49 × 10^6^	3.945	<0.001	[6.86 × 10^6^, 2.07 × 10^7^]
Prevalence	1.12 × 10^7^	3.67 × 10^6^	3.048	0.003	[3.92 × 10^6^, 1.85 × 10^7^]

Note: 95% CI, 95% Confidence Interval.

### Effects Regression Analysis

**Fig. 3.**
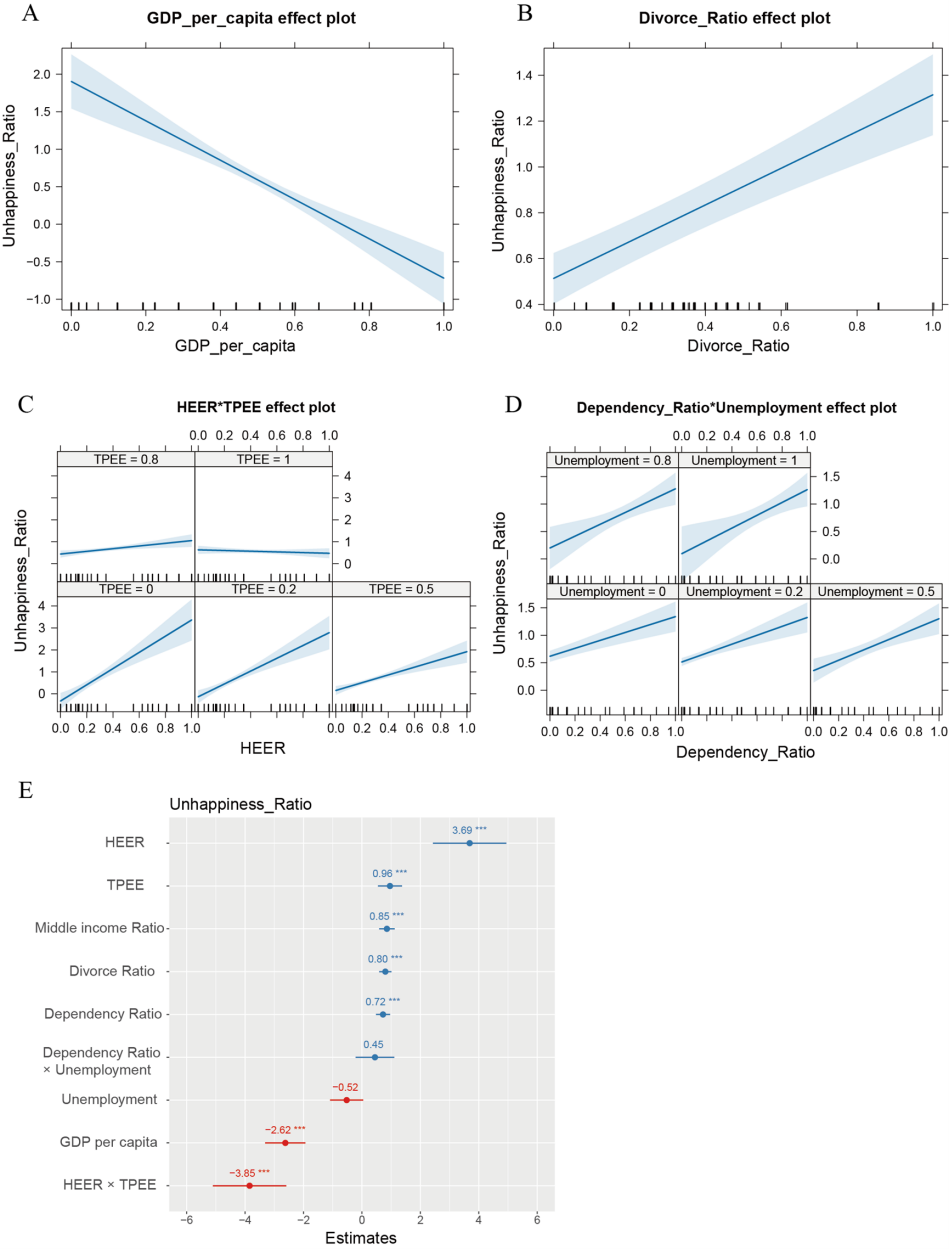
**Fixed and interaction effects from effects regression on the unhappiness ratio**. (A) Effect of GDP per capita. (B) Effect of dependency ratio. (C) Interaction between HEER and TPEE, demonstrating the moderating role of education spending. (D) Interaction between the dependency ratio and unemployment rate, illustrating an amplified risk with high unemployment rates. (E) Coefficient magnitudes of fixed effects, highlighting HEER as the strongest predictor (β = 3.69, *p* < 0.001). Data: 2003–2021.Note: *p < 0.05; ***p < 0.001; GDP, Gross domestic product; HEER, Higher education enrolment rate; TPEE, Total public expenditure on education.

A mixed-effects regression model was constructed to assess the fixed effects of key social determinants on the unhappiness ratio. Residual plots confirmed normality and homoscedasticity assumptions (**Supplementary Fig. 2A–D**). Increased GDP per capita was significantly associated with a decreased unhappiness ratio β = −0.312, *p* < 0.01, Fig. 3A), and a high dependency ratio was associated with an increased unhappiness ratio (β = 0.289, *p* < 0.05, Fig. 3B). Interaction terms demonstrated that when public education expenditure was low, high HEER was positively associated with unhappiness; by contrast, increased education spending attenuated this effect (Fig. 3C). The dependency ratio × unemployment interaction amplified unhappiness when unemployment exceeded 0.5 (Fig. 3D). Coefficient plots identified HEER as having the most influential fixed effect on unhappiness (β = 3.69, *p* < 0.001, Fig. 3E).

### Lagged Effect Analyses

ARDL analysis revealed that HEER had consistently negative short-term (lag 1) effects on depression incidence. The effect was marginally significant in 2016 (β = −15,820.0, *p* = 0.053) and became statistically significant from 2017 onwards, including 2017 (β = −15,837.6, *p* = 0.038), 2018 (β = −15,650.4, *p* = 0.026), 2019 (β = −15,206.0, *p* = 0.018) and 2020 (β = −8841.0, *p* = 0.001). Meanwhile, lag 2 and lag 3 effects remained consistently nonsignificant, indicating that educational competition mainly operates through short-term effects (Table 3).

**Table 3.** **Lagged effects of HEER on depression incidence**.

Year	Variable	Estimate	Std Error	t	*p*
2016	Intercept	1088433.0	143633.0	7.578	<0.001***
	education_index_lag1	−15820.0	8058.0	−1.963	0.053
	education_index_lag2	219.0	10939.0	0.020	0.984
	education_index_lag3	7734.0	8190.0	0.944	0.348
2017	Intercept	1099208.8	127498.3	8.621	<0.001***
	education_index_lag1	−15837.6	7492.8	−2.114	0.038*
	education_index_lag2	168.4	10239.4	0.016	0.987
	education_index_lag3	7259.8	7573.8	0.959	0.341
2018	Intercept	1101780.2	116335.3	9.471	<0.001
	education_index_lag1	−15650.4	6916.9	−2.263	0.026*
	education_index_lag2	468.9	9488.5	0.049	0.961
	education_index_lag3	6573.6	6971.9	0.943	0.348
2019	Intercept	1092524.0	104345.0	10.470	<0.001***
	education_index_lag1	−15206.0	6322.0	−2.405	0.018*
	education_index_lag2	6562.0	6371.0	1.030	0.306
2020	Intercept	1092718.0	93851.0	11.640	<0.001***
	education_index_lag1	−8841.0	2519.0	−3.510	<0.001***

Note: *p < 0.05; ***p < 0.001. HEER, Higher education enrolment rate.

The short-term (lag 1) effects of CHE per capita (medical_index) on depression incidence were also significant. In the early period, the effects were marginally significant in 2014 (β = −1627.3, *p* = 0.099) and 2015 (β = −1602.8, *p* = 0.070), indicating a weak immediate impact. From 2016 onwards, the negative effects became increasingly significant, including in 2016 (β = −1628.0, *p* = 0.055), 2017 (β = −1578.6, *p* = 0.035), 2018 (β = −1463.2, *p* = 0.021), 2019 (β = −1406.9, *p* = 0.015) and 2020 (β = −821.3, *p* < 0.001), suggesting that the short-term policy-sensitive effects became progressively stronger. Lag 2 and lag 3 terms were generally nonsignificant, indicating the dominance of short-term impacts (Table 4).

**Table 4.** **Lagged effects of per capita health expenditure on depression incidence**.

Year	Variable	Estimate	Std Error	t	*p*
2014	Intercept	1031768.6	111467.7	9.256	<0.001***
	medical_index_lag1	−1627.3	973.1	−1.672	0.099
	medical_index_lag2	8.3	1325.2	0.006	0.995
	medical_index_lag3	902.4	988.9	0.912	0.365
2015	Intercept	1035822.8	102500.5	10.106	<0.001***
	medical_index_lag1	−1602.8	870.9	−1.840	0.073
	medical_index_lag2	10.1	1191.0	0.008	0.993
	medical_index_lag3	813.5	881.8	0.923	0.359
2016	Intercept	1045490.3	95698.7	10.925	<0.001***
	medical_index_lag1	−1628.0	836.8	−1.946	0.055
	medical_index_lag2	−99.1	1148.5	−0.086	0.932
	medical_index_lag3	867.6	842.9	1.029	0.307
2017	Intercept	1043983.2	90788.0	11.499	<0.001***
	medical_index_lag1	−1578.6	736.9	−2.142	0.035*
	medical_index_lag2	73.4	1013.4	0.072	0.942
	medical_index_lag3	629.0	742.3	0.847	0.399
2018	Intercept	1030374.5	85694.7	12.024	<0.001***
	medical_index_lag1	−1463.2	622.4	−2.351	0.021*
	medical_index_lag2	140.0	856.3	0.164	0.871
	medical_index_lag3	498.7	629.1	0.793	0.431
2019	Intercept	1016185.0	78336.8	12.972	<0.001***
	medical_index_lag1	−1406.9	569.1	−2.472	0.015*
	medical_index_lag2	604.7	574.2	1.053	0.295
2020	Intercept	1015157.1	72075.7	14.085	<0.001***
	medical_index_lag1	−821.3	225.1	−3.648	<0.001***

Note: *p < 0.05; ***p < 0.001.

Similarly, the short-term (lag 1) effects of urban population/development on depression incidence showed a consistent negative association. The effects were borderline significant in 2015 (β = −33,407, *p* = 0.086) and 2016 (β = −33,291, *p* = 0.060) and became statistically significant from 2017 to 2020: 2017 (β = −32,981.4, *p* = 0.042), 2018 (β = −32,528.9, *p* = 0.029), 2019 (β = −31,414, *p* = 0.023) and 2020 (β = −821.3, *p* < 0.001), indicating the immediate effects of urban development on mental health outcomes. Lag 2 and lag 3 effects were consistently nonsignificant across all years (Table 5).

**Table 5.** **Lagged effects of urban population on depression incidence**.

Year	Variable	Estimate	Std Error	t	*p*
2015	Intercept	1964910.0	546357.0	3.596	<0.001***
	Urban_population_index_lag1	−33407.0	19206.0	−1.739	0.086
	Urban_population_index_lag2	−1868.0	25870.0	−0.072	0.943
	Urban_population_index_lag3	12715.0	19158.0	0.664	0.509
2016	Intercept	1980600.0	483509.0	4.096	<0.001***
	urban_development_index_lag1	−33291.0	17466.0	−1.906	0.060
	urban_development_index_lag2	−1314.0	23616.0	−0.056	0.956
	urban_development_index_lag3	11628.0	17425.0	0.667	0.507
2017	Intercept	1980241.2	431300.6	4.591	<0.001***
	urban_development_index_lag1	−32981.4	15967.0	−2.066	0.042*
	urban_development_index_lag2	−844.6	21660.3	−0.039	0.969
	urban_development_index_lag3	10794.7	15931.6	0.678	0.052
2018	Intercept	1967991.3	387596.6	5.077	<0.001***
	urban_development_index_lag1	−32528.9	14684.5	−2.215	0.029*
	urban_development_index_lag2	−478.9	19977.4	−0.024	0.981
	urban_development_index_lag3	10187.4	14652.7	0.695	0.489
2019	Intercept	1901252.0	337210.0	5.638	<0.001***
	urban_development_index_lag1	−31414.0	13640.0	−2.303	0.023*
	urban_development_index_lag2	9731.0	13628.0	0.714	0.477
2020	Intercept	1015157.1	72075.7	14.085	<0.001***
	urban_development_index_lag1	−821.3	225.1	−3.648	<0.001***

Note: *p < 0.05; ***p < 0.001.

### Gender- and Age-Stratified Analyses

**Fig. 4.**
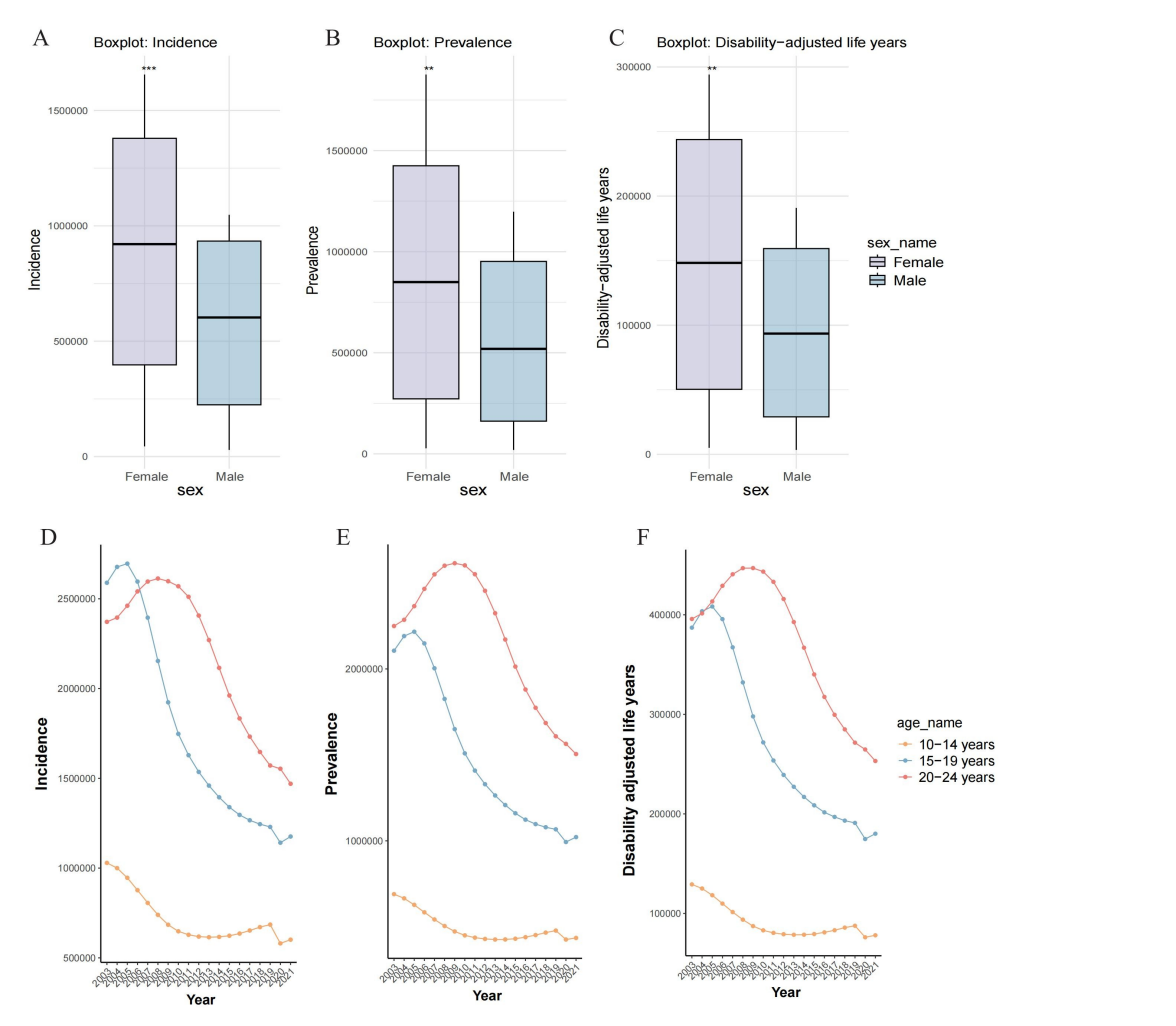
**Depression burden stratified by gender and age group**. (A–C) Distributions of depression incidence, prevalence and DALYs by gender, showing significantly higher burden in females (*p* < 0.001). (D–F) Age-stratified distributions across 10- to 14-, 15- to 19- and 20- to 24-year-olds, highlighting rising burden amongst 10- to 14-year-olds post-2017 and faster decline in 15- to 19-year-olds after 2010. Data sources: GBD. *p < 0.05; ***p < 0.001.

Gender-stratified analyses revealed significantly higher depression burden amongst females than males for incidence, prevalence and DALYs (*p* < 0.001, Fig. 4A–C), which may be partly related to sociocultural and developmental factors that differentially affect female adolescents. Age-stratified analyses showed that individuals aged 10–14 years had a lower depression burden than those aged 15–24 years (Fig. 4D–F). After 2010, the decline in depression burden was more pronounced amongst individuals aged 15–19 years than amongst aged 20–24 years, whereas incidence and DALYs amongst 10- to 14-year-olds exhibited a gradual upward trend.

**Fig. 5.**
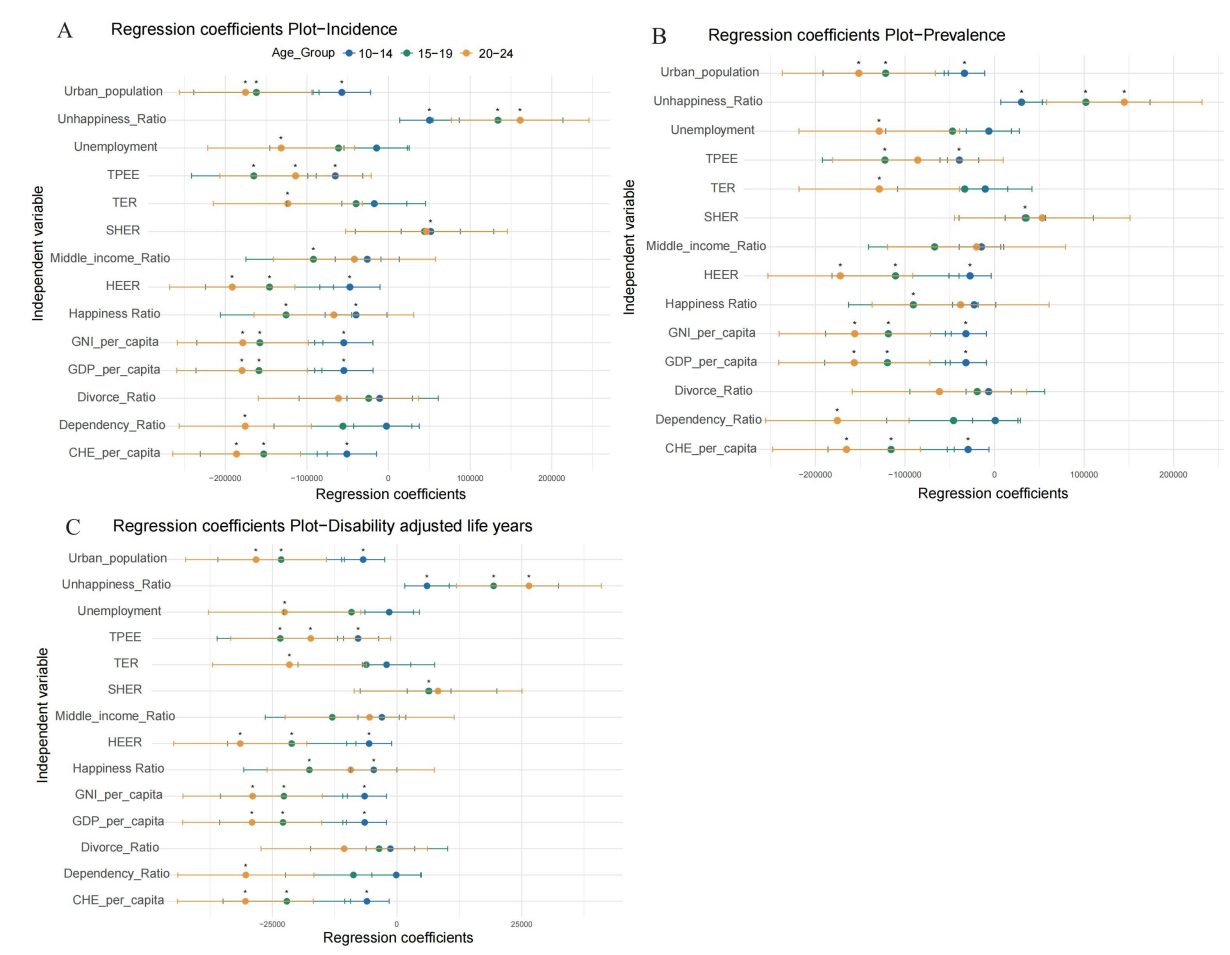
**Univariate regression of depression burden by age group**. (A) Regression coefficients (β) for social determinants predicting depression incidence. (B) Regression results for prevalence. (C) Regression results for DALYs. Key findings: Public education expenditure and urbanisation are negatively associated with the burden in 10- to 19-year-olds; HEER and CHE per capita are protective in 20- to 24-year-olds. Data: 2003–2021. *p < 0.05.

Univariate regression analyses by age group indicated that amongst individuals aged 10–19 years, TPEE and urban population percentage were significantly and negatively associated with depression burden (standardised β = −0.320, *p* < 0.05). By contrast, among those aged 20–24 years, HEER and CHE per capita were identified as protective factors (Fig. 5A–C). Additionally, the dependency ratio showed a significant negative association with depression prevalence in individuals aged 20–24 years (standardised β = −0.276, *p* < 0.05), suggesting that stronger family support structures may play a buffering role in this age group.

HEER was positively associated with depression burden amongst females aged 15–19 and 20–24 years. This association may reflect underlying mechanisms related to educational experiences and psychosocial stress. However, given the ecological nature of the data, these findings should be interpreted with caution, and further studies are needed to clarify the potential roles of educational pressure and gender-related factors [26, 27].

## Discussion

Recent research increasingly underscores the critical role of SDHs in adolescent depression [10]. Consistent with previous studies, our analysis confirmed that family-related factors—such as parental depression, low parental education and single-parent households—are strongly associated with heightened depression burden amongst adolescents [4, 13, 14]. Additionally, disrupted parenting styles and family conflict, particularly affecting female adolescents, contribute substantially to depression risk [28]. Our findings demonstrate significantly higher depression burden in female adolescents than in males, highlighting the importance of implementing gender-stratified mental health monitoring systems, strengthening social support structures and promoting gender equity policies to reduce mental health disparities.

Socioeconomic status emerged as another critical determinant of adolescent depression. Our mixed-effects analysis identified GDP per capita, urban population, and CHE per capita as significant protective factors against depression burden, indicating that economic growth, urban development and improved healthcare access may mitigate adolescent mental health issues. This finding aligns with previous research showing that high socioeconomic status correlates with increased perceived social support and positive emotions, leading to reduced depression risk [12]. Conversely, adolescents from rural or low-income backgrounds may experience reduced access to social support, contributing to elevated depression burden [29]. The study also observed that HEER is correlated positively with unhappiness, suggesting that intensified competition in educational environments can exacerbate psychological distress amongst adolescents.

Our results underscore that the depression burden amongst young Chinese people is intricately linked to multidimensional social environmental factors, showing age-stratified differences in vulnerability. Younger adolescents (10–19 years) were greatly influenced by family dynamics, educational opportunities and living conditions, reflecting their reliance on parental support and stable social environments—a finding consistent with Hua Xu’s work [25]. Conversely, young adults (20–24 years) exhibited great sensitivity to external societal stressors, including educational competition, urban pressures and limited access to healthcare, aligning with the literature indicating that personal aspirations and societal expectations increasingly shape psychological well-being during late adolescence [30]. Protective social support from family, friends and teachers remains essential in buffering individuals against these stressors across various developmental stages [31].

The findings highlight the potential importance of age-stratified, targeted interventions, although causal relationships cannot be inferred from this study. For younger adolescents, the observed associations suggest that enhancing parental mental health literacy, promoting positive parent–child interactions and supporting family-centred programs could be beneficial. Schools may also contribute through teacher training aimed at identifying early signs of psychological distress. For young adults, the results indicate that stress related to competition and perfectionism may be relevant, pointing to possible benefits of peer-support groups, career counselling and youth-focused services, particularly in urban areas where family support may be limited [32]. Additionally, the socioeconomic disparities observed in the study suggest that targeted scholarships or subsidies might help mitigate inequalities in mental health outcomes [29].

Our lagged effect analyses revealed that HEER, CHE per capita and urbanisation exert significant short-term lagged impacts on depression burden, supporting the hypothesis that structural social determinants have cumulative and immediate effects on mental health trajectories [11]. For instance, increased educational investment may reduce the incidence of depression after improvements in educational infrastructure and staff capacity, although these effects appear transient, underscoring the necessity for sustained and flexible policy responses [33].

The inclusion of HEER × TPEE and unemployment × dependency ratio interactions highlight the importance of contextual moderators in shaping adolescent depression burden. Rising HEER may intensify competition. However, this effect appears to be buffered when TPEE increases. This pattern is consistent with the competition–resource matching mechanism described in prior studies. Similarly, the impact of unemployment may be amplified in households with high dependency ratios. In such contexts, economic strain is compounded by caregiving responsibilities. This observation aligns with socioecological models, which emphasise family structure as an important moderator of social stressors. Overall, these findings suggest that adolescent mental health is influenced not only by individual social determinants but also by their combined and interacting effects. This phenomenon highlights the importance of policy approaches that integrate resource allocation and family support structures.

Although the unhappiness ratio demonstrated significant associations with depression burden indicators in our univariate analyses, its interpretation requires caution. Unhappiness is a subjective measure of well being rather than a clinically validated diagnostic indicator of depression. Prior research on the GBD methodology emphasises that depression burden estimates are derived from standardised psychiatric instruments such as the Composite International Diagnostic Interview or Patient Health Questionnaire, not from self reported happiness metrics. Subjective well being indicators are influenced by cultural norms, transient life events and reporting biases, which limit their reliability as proxies for clinical depression. Although the unhappiness ratio may capture aspects of psychosocial distress and provide exploratory insights into population level mood states, it should not be considered a robust substitute for validated measures of depression burden. Future studies should prioritise the use of standardised diagnostic tools and longitudinal clinical data to ensure accuracy, comparability and policy relevance in assessing adolescent mental health.

A methodological limitation of this study lies in the selection of variables for the ARDL models. HEER, CHE per capita and urbanisation were chosen because of their theoretical relevance to adolescent depression burden and data availability. However, the absence of a formal prior theoretical framework or systematic model selection procedure means that these specifications may not fully capture the complexity of social determinants. Although the chosen variables align with socioecological perspectives—reflecting educational competition, public health investment and structural changes in social support environments—the reliance on literature review and pragmatic data constraints rather than a structured screening process limits the robustness of the ARDL specification. Future research should employ systematic approaches, such as information criteria, stepwise selection or Bayesian model comparison, to strengthen methodological rigor and ensure consistency with established theoretical frameworks. Our findings advocate for a comprehensive, multilevel policy framework integrating age- and gender-specific risks into national adolescent mental health strategies. Policymakers should adopt a socioecological perspective to intervene across family, school and community contexts. Longitudinal data collection is crucial for tracking the evolution of social determinants and informing timely interventions.

## Conclusions

This nationwide longitudinal study demonstrates that despite the decline in overall depression burden amongst Chinese adolescents aged 10–24 years from 2003 to 2021, a notable increase has emerged in the 10- to 14-year age group since 2017. Socioenvironmental factors exerted significant and age-stratified effects: Economic development, urbanisation and health expenditure acted as protective factors. By contrast, HEER, which reflects educational competition, was associated with increased burden, particularly among females aged 15–24 years. Additionally, these factors showed short-term lagged effects, and interaction analyses indicated that public education expenditure could mitigate the adverse impact of educational competition.

Overall, the findings highlight substantial age and gender heterogeneity in the social determinants of adolescent depression, underscoring the need for stratified and targeted mental health policies, with priority attention to early adolescents and female populations.

## Availability of Data and Materials

The datasets used and/or analysed during the current study were available from the corresponding authors on reasonable request.

## Supplementary Material

Supplementary material associated with this article can be found, in the online version, at https://doi.org/10.62641/aep.v54i3.2236.
