# Supplementary material for: Age-Stratified Analysis of Social Environmental Drivers of Depression Among Chinese Young People Aged 10–24 Years
Source: Actas Esp Psiquiatr. 2026 Jun 15;54(3):663–79. doi: 10.62641/aep.v54i3.2236 (PMC13294762; doi:10.62641/aep.v54i3.2236)
Supplement: Supplementary file 1 [file ActEsp-54-3-663-679-s1.docx]

**Supplementary Figure Legends:**

**Figure S1. Correlation Matrix of Independent Social Determinants.**

**Figure S2. Residual Diagnostics for Mixed-Effects Model on Unhappiness.**

**Supplementary Table Legends:**

**Table S1.** Univariate Regression Between Unhappiness Ratio and Social Environmental Factors


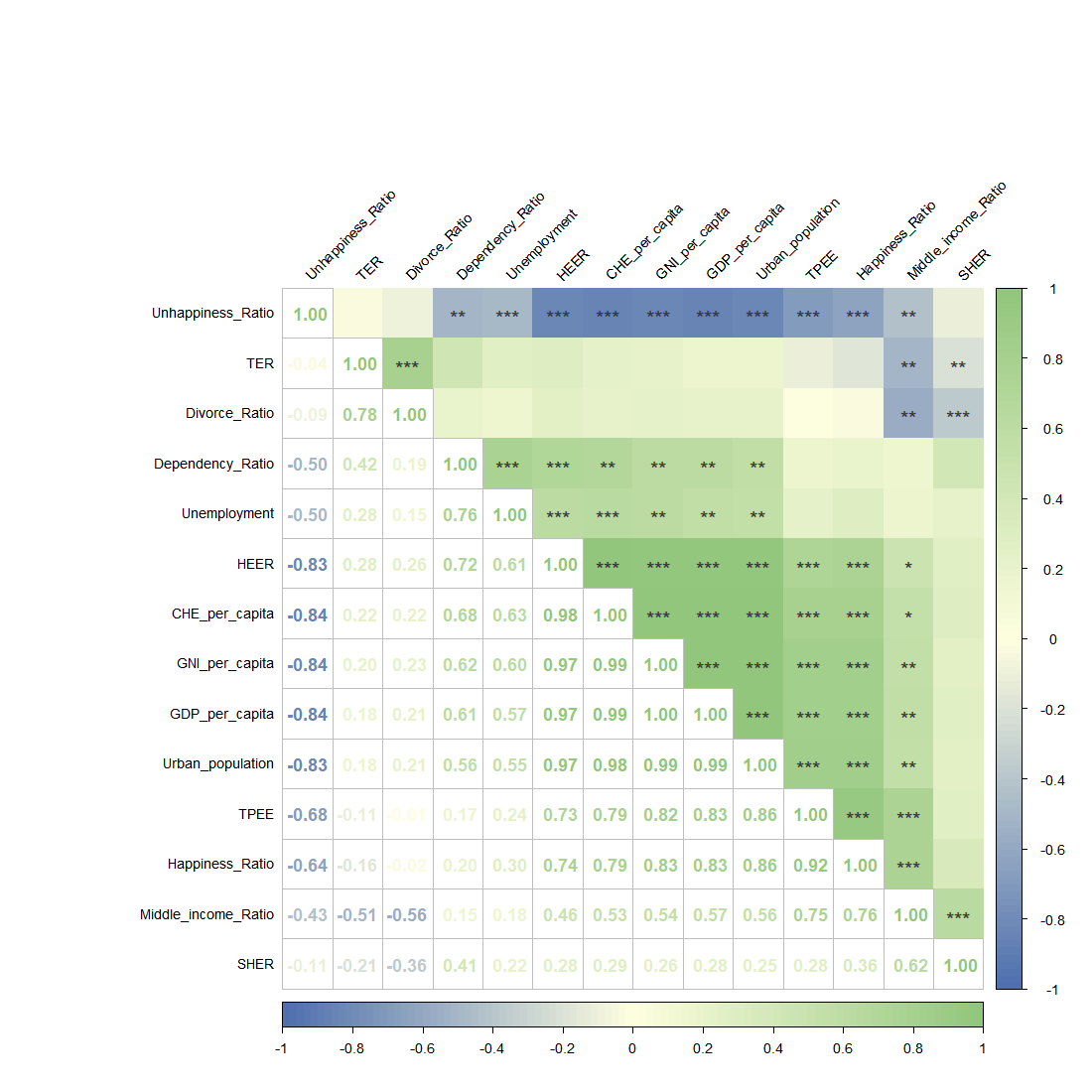


**Figure S1. Correlation Matrix of Independent Social Determinants.**

Displays pairwise Pearson correlation coefficients among macro-level social variables (GDP per capita, GNI per capita, CHE, HEER, urban population, TPEE, and dependency ratio) from 2003 to 2021. Strong correlations (|r| > 0.7) indicate the presence of multicollinearity, which requires adjustment in regression models. Data sources: World Bank, CGSS.

Note: TER: Total education level of respondent; HEER: Higher education enrollment rate; CHE: Per capita health expenditure; GNI: Gross national income; TPEE: Total public education expenditure; SHER: Spouse/partner's highest education rating.


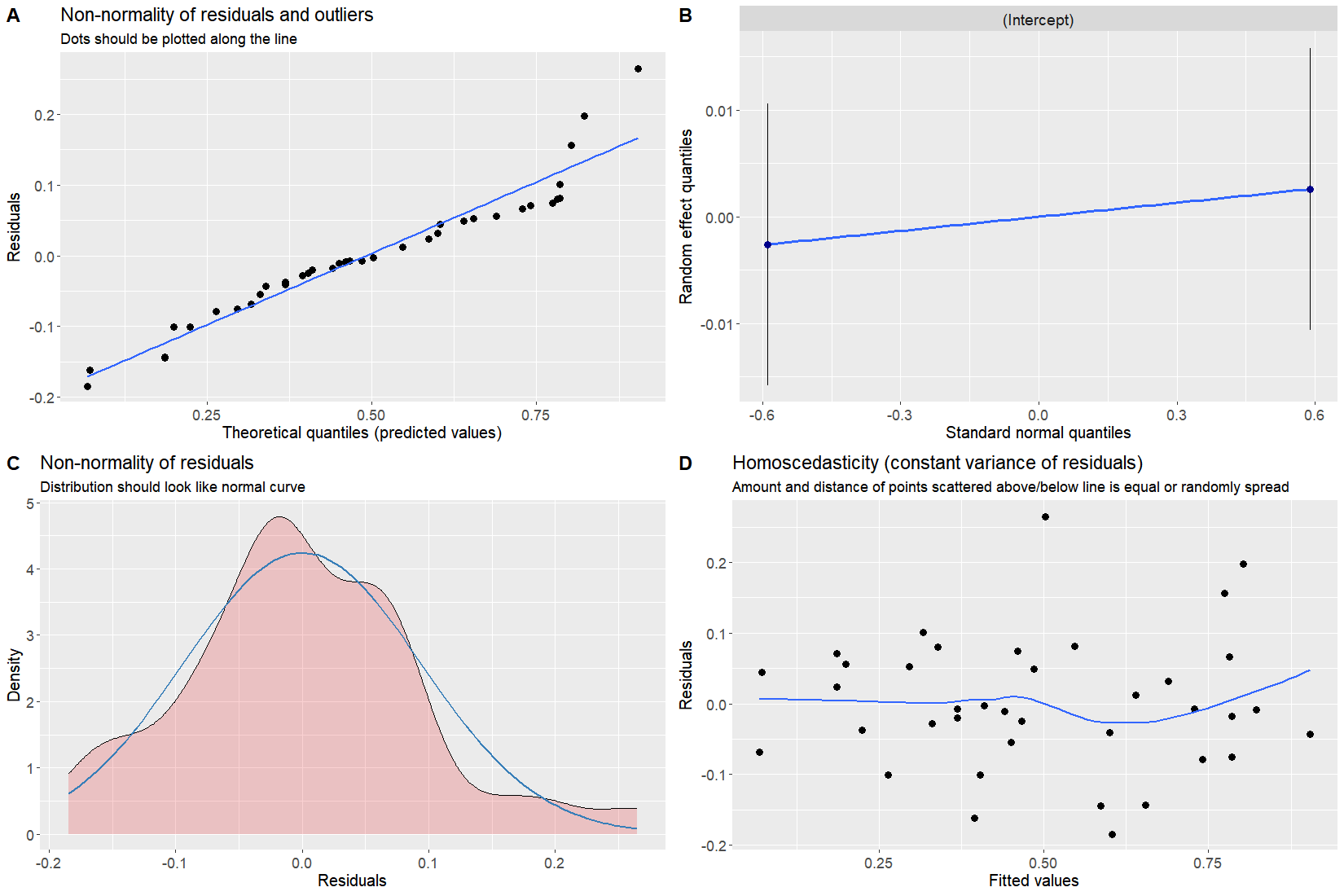


**Figure S2. Residual Diagnostics for Mixed-Effects Model on Unhappiness.**

(A) Residuals vs. theoretical quantiles (Q-Q plot) to assess normality. (B) Random effect of gender plotted against standard normal quantiles. (C) Histogram of residuals verifying approximate normal distribution. (D) Residuals vs. fitted values examining homoscedasticity. Data: 2003–2021 mixed-effects regression on unhappiness ratio with gender as a random effect.

**Table S1. Univariate Regression Between Unhappiness Ratio and Social Environmental Factors**

| **Dependent variable** | **Independent variable** | **coefficient** | **P** |
| --- | --- | --- | --- |
| Unhappiness Ratio | Unemployment | -0.498 | <0.001 |
| Unhappiness Ratio | GDP per capita | -0.841 | <0.001 |
| Unhappiness Ratio | GNI per capita | -0.838 | <0.001 |
| Unhappiness Ratio | CHE per capita | -0.842 | <0.001 |
| Unhappiness Ratio | Urban population | -0.834 | <0.001 |
| Unhappiness Ratio | HEER | -0.825 | <0.001 |
| Unhappiness Ratio | TPEE | -0.680 | <0.001 |
| Unhappiness Ratio | Dependency Ratio | -0.504 | <0.001 |
| Unhappiness Ratio | Divorce Ratio | -0.086 | 0.365 |
| Unhappiness Ratio | TER | -0.035 | 0.711 |
| Unhappiness Ratio | SHER | -0.113 | 0.230 |
| Unhappiness Ratio | Happiness Ratio | -0.637 | <0.001 |
| Unhappiness Ratio | Middle income Ratio | -0.434 | <0.001 |

Note: GNI: Gross national income; CHE:Per capita health expenditure; HEER: Higher education enrollment rate; TPEE: Total public expenditure on education; TER: Total education level of respondent; SHER: Spouse/partner's highest education rating.
